# Tailoring magnetic anisotropy gradients by ion bombardment for domain wall positioning in magnetic multilayers with perpendicular anisotropy

**DOI:** 10.1186/1556-276X-9-395

**Published:** 2014-08-13

**Authors:** Michał Matczak, Bogdan Szymański, Piotr Kuświk, Maciej Urbaniak, Feliks Stobiecki, Zbigniew Kurant, Andrzej Maziewski, Daniel Lengemann, Arno Ehresmann

**Affiliations:** 1 Institute of Molecular Physics, Polish Academy of Sciences, ul. M. Smoluchowskiego 17, Poznań 60-179, Poland; 2 NanoBioMedical Centre, Adam Mickiewicz University, ul. Umultowska 85, Poznań 61-614, Poland; 3 Laboratory of Magnetism, Faculty of Physics, University of Białystok, ul. Lipowa 41, Białystok 15-624, Poland; 4 Department of Physics, University of Kassel, Heinrich-Plett-Str. 40, Kassel 34132, Germany; 5 Center for Interdisciplinary Nanostructure Science and Technology (CINSaT), University of Kassel, Heinrich-Plett-Str. 40, Kassel 34132, Germany

**Keywords:** Perpendicular magnetic anisotropy, Ion bombardment, Domain walls

## Abstract

Graded anisotropy magnetic materials possess a coercive field changing laterally with position. A simple fabrication procedure to produce such an anisotropy gradient in a polycrystalline Au/Co layer system without lateral thickness variation and with perpendicular magnetic anisotropy, prototypical for a large variety of thin film systems, is shown. The procedure uses light-ion bombardment without the use of a mask. Magnetization reversal in this polycrystalline layer system takes place by unidirectional movement of a single domain wall only in regions with larger anisotropies and anisotropy gradients. In this anisotropy/anisotropy gradient regime, the domain wall is oriented perpendicular to the coercive field gradient, and it can be positioned along the gradient by an appropriate magnetic field pulse. For smaller anisotropies/anisotropy gradients, the natural anisotropy fluctuations of the polycrystalline layer system induce magnetization reversal dominated by domain nucleation.

**PACS:**

75.30.Gw; 75.70.Cn; 75.60.Ch

## Background

Magnetic domains in uniform layers with perpendicular magnetic anisotropy (PMA) take various shapes (cylindrical, labyrinth, maze, dendritic) that indicate the lack of preferential direction within the plane of the layer (see, e.g., [1-4]). Furthermore, the propagation velocity of domain walls (DWs), in a uniform field perpendicular to the layer plane, is isotropic. In contrast, in films with a lateral PMA gradient, it has been shown that magnetization reversal in a perpendicular field may occur by unidirectional propagation of a DW parallel to the gradient of the coercive field (*H*_C_) towards positions with higher values of *H*_C_[3,5,6]. This feature has the potential to be used for a controllable motion of the DW by applying a uniform external field [6], for wall pinning at defined locations [7-9], or for wall injection [7], with a variety of applications, like, e.g., the transport of magnetic beads by the moving stray field trap of a moving DW [10-14], for fluid mixing by moving particles [11,12,14], in sensors registering maximum values of varying magnetic fields [15], or in mass memories of ‘shift register’ type [16,17]. If it is possible to fabricate a flat material system with an anisotropy gradient along one coordinate axis with a negligible gradient along the other lateral coordinate, a controlled displacement of a straight DW would become feasible by uniform magnetic field pulses, avoiding complicated external field configurations (fields with special gradients, crossed perpendicular and in-plane fields, see, e.g., [1]). For a controlled positioning of a straight DW by an external magnetic field, the range of anisotropy of the material system and the gradient along a given direction are the essential characteristics [5]. The requirements on these two parameters are different depending on whether a pinning at a precise position (in the nm range) of the DW is of key importance or whether the displacement of a single straight DW in a given direction over a relatively long distance (in the sub-mm range) is essential. A reproducible DW pinning occurs when the change of PMA takes place on a distance *δ* much shorter than the width *w* of the domain wall [5]. The pinning field *H*_pin_ is then proportional to the difference of anisotropies *K*_eff,1_, *K*_eff,2_ at the borders of the graded area: $\lim_{\delta \to 0}H_{\mathrm{pin}}=\left(K_{\mathrm{eff},1}-K_{\mathrm{eff},2}\right)/2\mu_{0}M_{s}$. If, however, the anisotropy gradient extends over a distance much larger than the DW width, the pinning field approaches zero. Nevertheless, a DW in such a system can be displaced for defined distances by pulses of a homogeneous external magnetic field, as was observed for (Co/Au)_3_ layers [6] possessing an anisotropy gradient over a distance larger than the DW width. In these latter experiments, however, there have been indications that the magnetization reversal in areas with weak PMA (small *H*_C_; Figure three in [6]) does not take place by propagation of a single DW only, but also by domain nucleation.

In the present contribution, we will first describe a simple method to fabricate the mentioned graded PMA in a polycrystalline Co/Au layer system by a special procedure of kiloelectronvolt light-ion bombardment subsequent to the deposition of the layer system. These prototype polycrystalline graded PMA layer systems are used to investigate how their natural PMA fluctuations influence the magnetization reversal, and under which conditions, the unidirectional propagation of a domain wall is observed. The propagation of DW is investigated for increasing value of magnetic field pulses of 1-s duration.

## Methods

**Figure 1:**
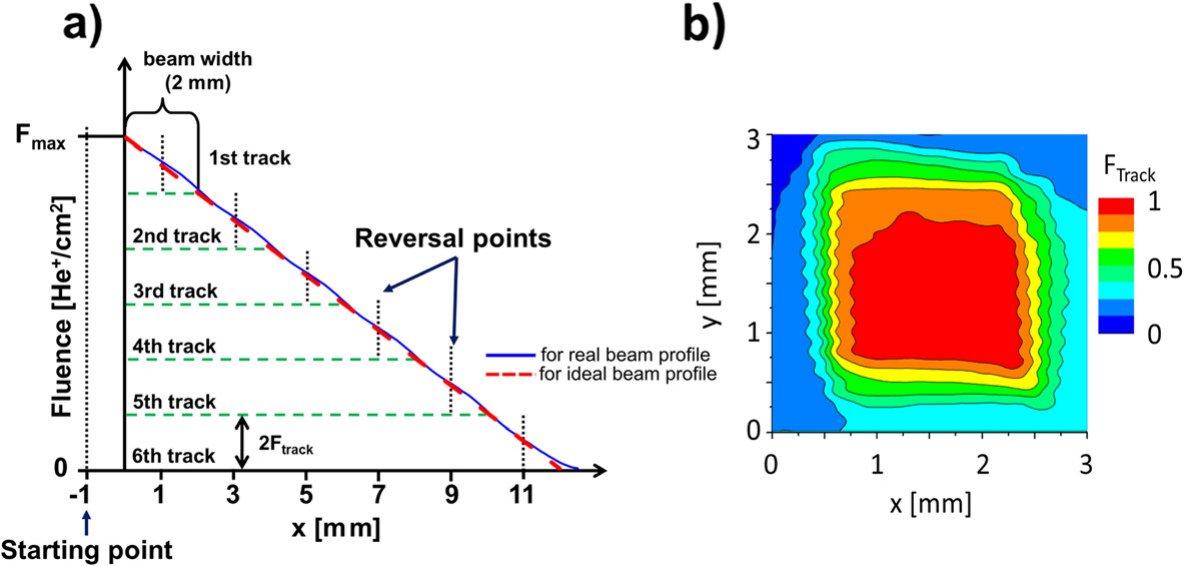
**Fabrication of the monotonically varying ion fluence with defined ion beam profile. (a)** Sketch of the ion bombardment procedure for constant fluence gradient along the *x*-direction. The cumulative fluence *F* versus *x* position after six tracks of an ion beam with the idealized rectangular intensity cross-section (dashed line) and the experimental intensity cross-section (solid line) of the beam profile shown in **(b)**. The ‘wavy’ edges of the beam intensity at *x* ≈ 0.5 and 2.5 are artifacts caused by measurement procedure (in which a Faraday cup with an opening of 0.1-mm diameter scans the beam along the *x*-direction).

Anisotropy gradients in layered film systems (e.g., of the Pt/Co or Au/Co type) in which PMA results from a strong surface anisotropy may be obtained by the continuous variation of the ferromagnetic layer thickness [3], i.e., by a wedged ferromagnetic layer. For Co/Au layer systems, however, the Co layer thickness range for such a manipulation is restricted to thicknesses between about 0.6 and 1.2 nm. Therefore, achievable thickness gradients over macroscopic distances (e.g., in the mm range) are very small, and they are sensitive to the smallest fluctuations within the wedge deposition procedure. Alternatively, an anisotropy gradient can be fabricated by light-ion bombardment [18] with continuously increasing/decreasing the ion fluence along one lateral coordinate. For the present experiments, we chose this second strategy. The layer system was a prototype magnetron sputtered (Co^0.8 nm^/Au^1 nm^)_2_ film system deposited on 14.5 × 10 mm^2^ naturally oxidized Si(100) substrates with Ti^4 nm^/Au^60 nm^ buffer layers. The base pressure during sputtering was < 1 × 10^−6^ Pa with Ar process gas (Ar partial pressure approximately 10^−2^ Pa). The Co thickness *t*_Co_ was chosen to be well above the critical thickness where the Co layer becomes superpara- or paramagnetic after light-ion bombardment [19] and at the same time thin enough to preserve a large PMA. Moreover, *t*_Co_ selected here ensures that the magnetization reversal in the unbombarded Au/Co/Au system in a perpendicular magnetic field develops primarily by DW movement [2]. Additionally *t*_Au_ was chosen to couple the Co layers ferromagnetically for a simultaneous reversal of both ferromagnetic layers by a synchronized motion of the DWs. For fabrication of the graded anisotropy by light-ion (10-keV He^+^ ions) bombardment, a monotonically varying ion fluence along a given lateral direction is essential. In the past, a wedged ion stopper layer was used [6]. However, a wedged stopper layer cannot be removed easily after deposition, being disadvantageous for some applications like bead transport through stray fields of moving DWs, where the distance between the magnetic layers and the bead and thus the stray field characteristics changes along the wedge. Therefore, a monotonical ion flux variation must be realized by a monotonical change of the time the ion beam resides over a certain sample area at constant ion flux. The used method is schematically explained in Figure 1 for an ion beam with an idealized fluence cross-section being constant within a rectangle of 2 × 2 mm^2^ and zero elsewhere:

Move the sample to a position where the ion beam just misses the sample. The position of the ion beam center *x*_cb_ in a coordinate system with *x* = 0 defining the left edge of the sample is then at *x*_cb_ = −1 mm. Move then the computer-controlled sample stage along the − *x*-direction at constant velocity and reverse the movement, when the ion beam center is at *x*_cb_ = +1 mm, i.e., at a position when the 2 × 2 mm^2^ ion beam bombards the whole *x* range from *x* = 0 to 2 mm. Move again the sample backward with a constant velocity to the starting position. With this first track, a constant fluence gradient d*F*/d*x* along the *x*-direction within the first 2 mm of the sample is achieved. After this first track, move the sample stage relative to the ion beam center from its starting position (*x*_cb_ = −1 mm) to *x*_cb_ = +3 mm where the movement again is reversed. The reversal point is chosen such that it is displaced relative to the reversal point of the first track by one width of the ion beam. The stage movement of the second track adds a constant fluence of 2 × *F*_track_ to the already existing gradient within the first 2 mm in the *x*-direction and causes the same gradient in the *x*-direction within the next 2 mm as the one fabricated in the first 2 mm by the first track. By subsequent tracks, increasing each time the travel of the stage by one width of the ion beam, the cumulative fluence applied to the sample results in a constant gradient in the *x*-direction within the bombarded stripe. The experiments were carried out with a home-built He^+^ ion source set to 10-kV acceleration voltage with low divergence [20]. A 2 × 2 mm^2^ square aperture cuts out a portion of the beam displaying an approximate square ion flux cross-section, with a plateau-like flux *j*(*x*, *y*) = d*N*(*x*, *y*)/(d*t* · d*A*) (*N* = number of He ions, *t* = time, *A* = area through which the He ions flowed) in the middle of the square and decreasing flux at the four edges of the square (Figure 1b). Two stripes (stripe 1 and stripe 2) were bombarded with two different plateau fluxes *j* applying the sample movement scheme described above. Fluence profiles *F*(*x*,*y*) = ∫*j*(*x*, *y*)d*t* were calculated for a 12-mm-long stripe which agreed, within experimental error, with that expected from the ideal rectangular profile. For both stripes, the relative fluence profiles *F*(*x*)/*F*_max_ are the same (Figure 1a). The areas of the two stripes were magnetically characterized at room temperature by polar magneto-optical Kerr effect (P-MOKE). Local hysteresis loops (diameter of laser spot on the sample surface - 0.5 mm) were measured with a magnetic field sweep rate of 1.4 (kA/m)/s. The measurements were performed as a function of the *x*-coordinate with the laser beam center in the center of the bombarded stripe. For comparison, loops of unbombarded areas were also measured. After the six tracks, the cumulated maximum fluence *F*_max_ for stripe 1 was 1.06 × 10^15^ He^+^/cm^2^ and for the second stripe 5.03 × 10^14^ He^+^/cm^2^. The stage velocity was chosen for an average fluence gradient of d*F*/d*x* = 8.8 × 10^13^ (He^+^/cm^2^)/mm for stripe 1 and 4.4 × 10^13^ (He^+^/cm^2^)/mm for stripe 2 and maximum fluences based on previous investigations [19,21]. Due to the flux decrease of the ion beam at the edges of the rectangle, also a fluence gradient along the *y*-direction is observed. This gradient changes with the *x*-coordinate between d*F*/d*y* (*x* = 0 mm) = 7.5 × 10^14^ (He^+^/cm^2^)/mm to d*F*/d*y* (*x* = 8 mm) = 2.5 × 10^14^ (He^+^/cm^2^)/mm. At small *x*-coordinates, gradients along the *y*-direction are roughly 1 order of magnitude larger as the ones along the *x*-direction.

## Results and discussion

**Figure 2:**
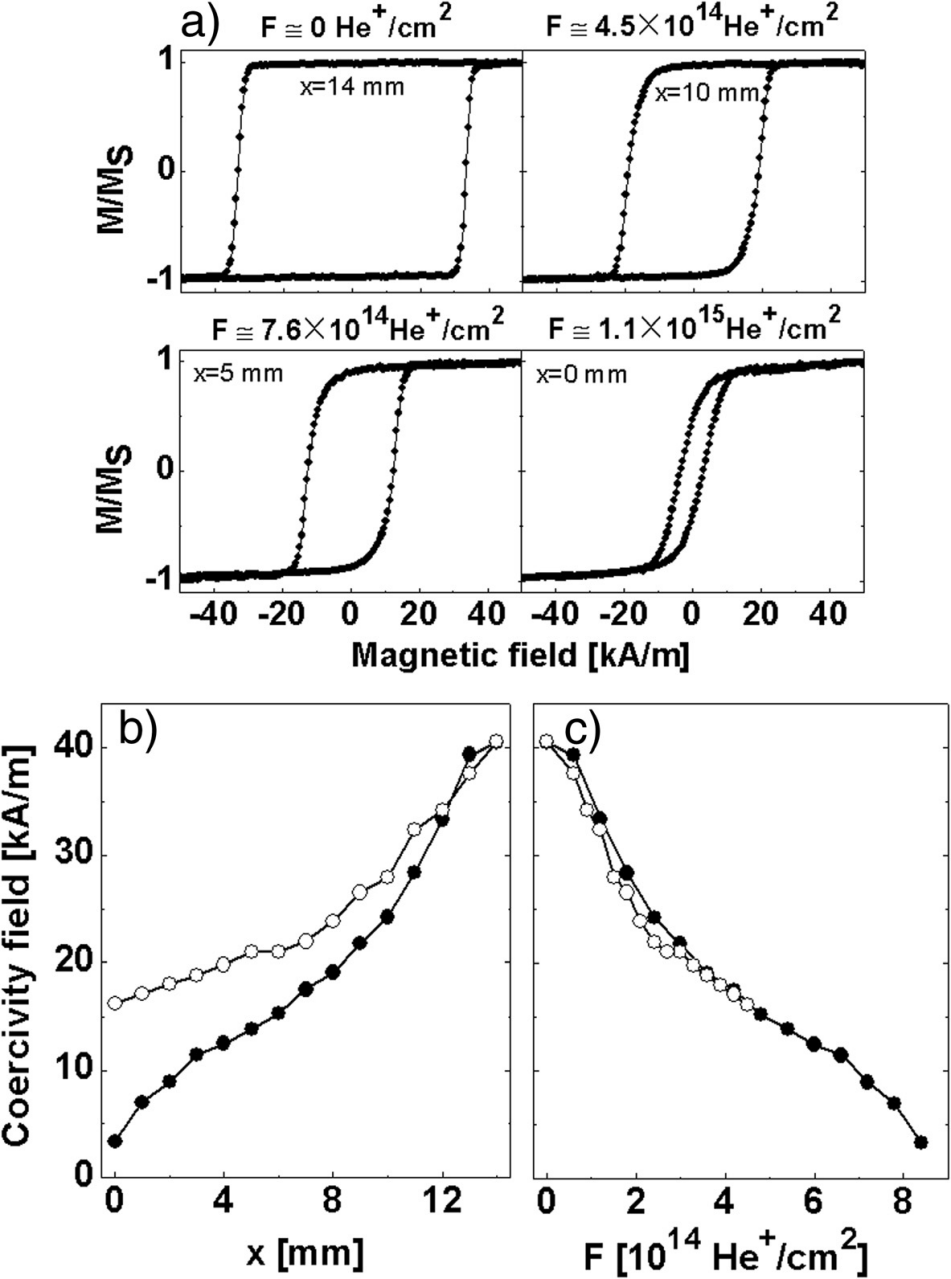
**The influence of ion bombardment with different fluences on coercive field. (a)** Representative hysteresis loops for stripe 1 taken along the *x*-axis. *H*_C_ as a function of the *x*-coordinate **(b)** and as a function of *F***(c)** (full circles - stripe 1, open circles - stripe 2). Note that the fluence gradient and coercive field gradient have opposite signs.

The as-deposited Co/Au multilayers with a cobalt thickness of *t*_Co_ = 0.8 nm show distinct perpendicular anisotropy and a rectangular hysteresis loop (Figure 2a). The magnetization reversal of the multilayers in perpendicular magnetic field (*H*_ext_) starts from a small number of nucleation centers and develops by DW motion, resulting in nearly circular domains similarly as was observed in ultrathin Au/Co/Au [22] and Pt/Co/Pt [23] films with significant PMA. Representative hysteresis loops for bombarded multilayers taken at different positions along the *x*-coordinate are also shown in Figure 2a. For low *F* (≤3 × 10^14^ He^+^/cm^2^), the loops stay rectangular and are only slightly rounded. This indicates that the DW motion remains the dominant mechanism of the magnetization reversal. For higher *F*, the loops become more rounded, which suggests that with increasing *F* (decreased anisotropy) domain nucleation becomes increasingly important. For *F* ≥ 6 × 10^14^ He^+^/cm^2^, the loop shapes deviate strongly from rectangular (at *H =* 0 *M*_R_/*M*_S_ < 1), which indicates significant spatial variations of nucleation fields on a length scale smaller than the probing area of the P-MOKE laser on the sample. For the highest fluences of *F* ≈ 1 × 10^15^ (He^+^/cm^2^), the surface contribution to the effective anisotropy is strongly reduced, and as a consequence, the shape anisotropy dominates resulting in easy-plane (parallel to the sample plane) anisotropy. The observed loop changes slightly differ from the results published for Si/SiN_*x*_/Pt^20 nm^/[Pt^1 nm^/Co^0.3 nm^]_10_/Pt^2 nm^ layer systems [24], where for homogeneous ion bombardment no changes of the loop squareness was observed (for fluences which reduce the coercive field to less than 90% of the value for the unexposed system). In Figure 2b, the measured *H*_C_ is plotted as a function of the *x*-position for both stripes. A monotonic decrease of coercive field with *F* is observed, which is caused by the reduction of the interface anisotropy due to modification of Co/Au and Au/Co interfaces through the increasing fluence of the 10-keV He^+^ ions [19,25,26]. Comparing the *H*_C_(*F*) and *H*_C_(*x*) dependencies for the two stripes with different gradients (Figure 2b,c), it can be seen that the *H*_C_(*F*) basically does not depend on d*F*/d*x*.

The measurement of hysteresis loops along the gradient in the *y*-direction (at the stripe border) has not been carried out as the observation area would encompass regions of significantly different *H*_C_.

**Figure 3:**
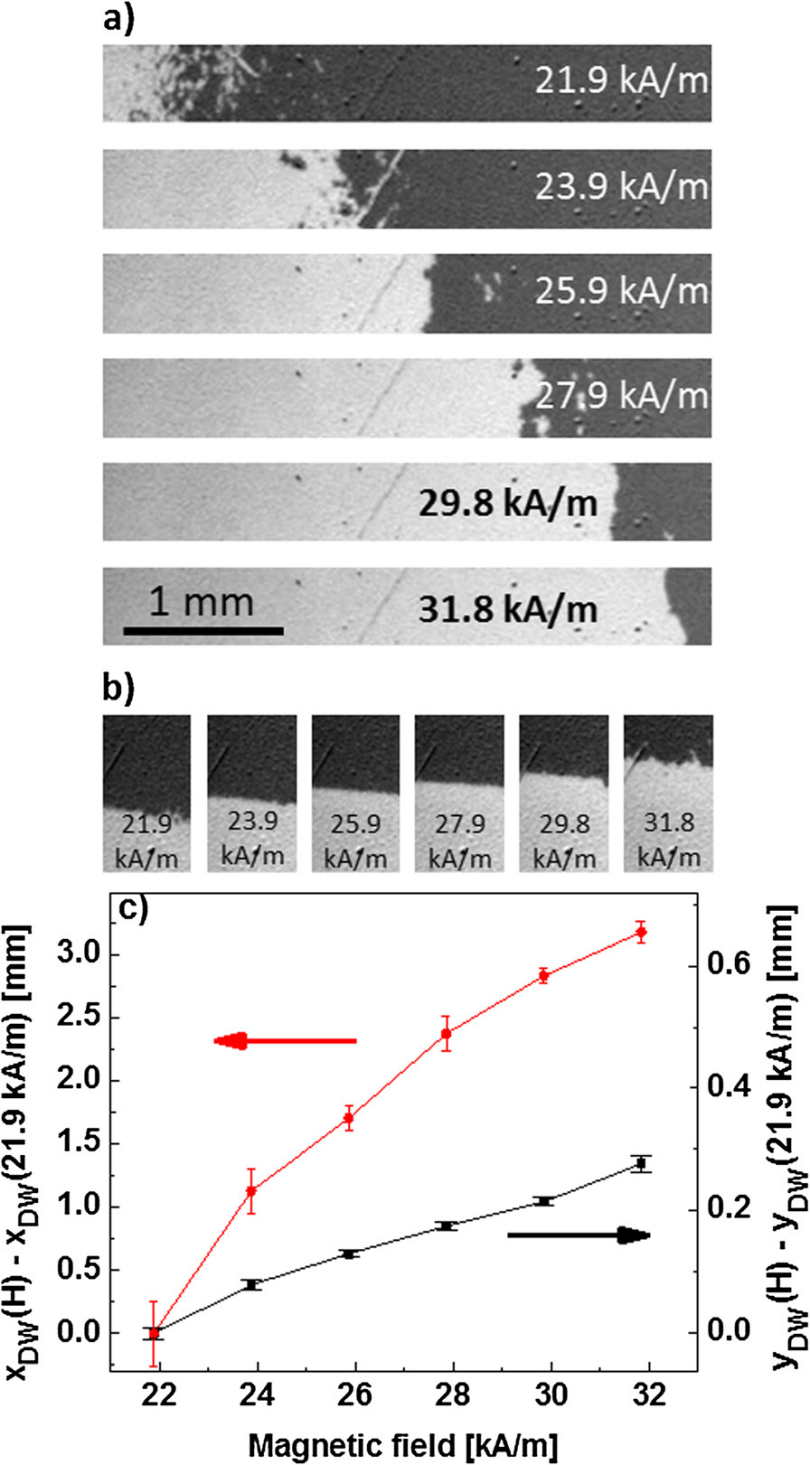
**Domain wall propagation for two different coercive field gradients.** Propagation of the DW in the region with small **(a)** and high **(b)** coercive field gradient, i.e., for areas A and B marked in Figure 4, respectively. **(c)** Positions of the DW [*x*_DW_(*H*_ext_) − *x*_DW_(*H*_ref_ = 21.9 kA/m)] and [*y*_DW_(*H*_ext_) − *y*_DW_(*H*_ref_ = 21.9 kA/m)] relative to its position for a reference field pulse of 21.9 kA/m as a function of field pulse amplitude *H*_ext_. The scales in (a) and (b) are the same.

The hysteresis loops in Figure 2 were measured with a slow field sweep rate of 1.4 (kA/m)/s, which correspond to the quasistatic limit. The position of the DW seen in the Kerr microscope for a given field pulse corresponds closely to the position of the sample where the coercive field of the loop equals that field (see Figures 2 and 3). It proves that for field change rates used here both methods give similar results. Thus, *H*_C_ is treated as a parameter which allows to approximately describe the reversal process measured under specific conditions.

The nonlinearity of *H*_C_(*x*) and *H*_C_(*F*) results probably from a distinct difference in the character of magnetization reversal in areas of small and large fluences (high and small *H*_C_ values) (see the discussion of Figure 3). It should be noted that the *K*_eff_ dependence on the fluence is not linear [24].

**Figure 4:**
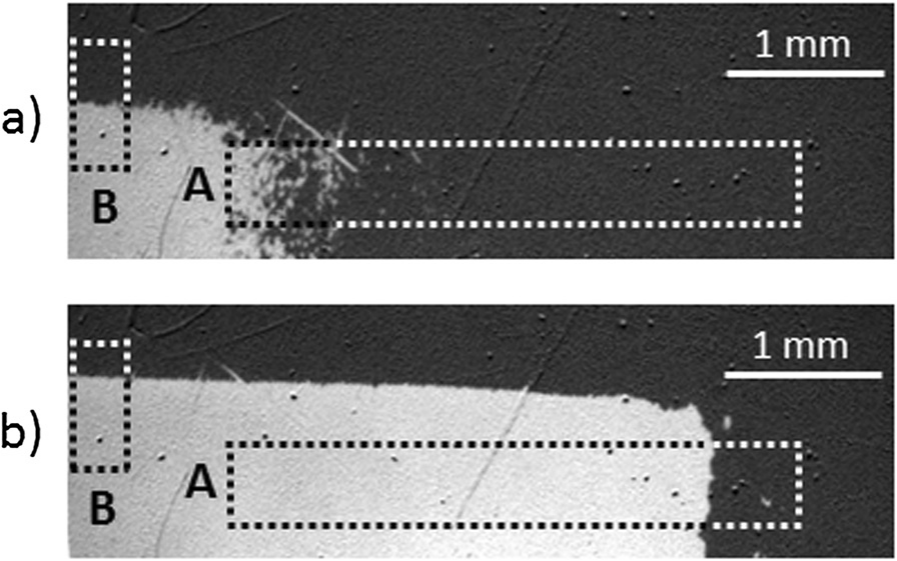
**P-MOKE images of the domain structure with coercive field gradient.** Domain structure of a representative area of the sample (a part of stripe 1) recorded after a field pulse of 21.9 and 29.8 kA/m for panels **(a)** and **(b)**, respectively. The evolution of DW positions in marked areas A and B is discussed with Figure 3.

The domain structure was observed using a MOKE microscope, collecting images at remanence (after the application of a magnetic field pulse) similarly as described in [6]. Figure 4a,b shows exemplary images of the domain structure visible within stripe 1 of the sample. Prior to magnetic characterization, the sample was magnetized up to saturation with −79.6 kA/m field (dark areas - *M* directed towards the substrate), a field much higher than the coercive field of the as-deposited multilayers. Subsequently, a 1-s-long pulse of a finite and oppositely directed *H*_ext_ was applied. In Figure 4a,b, the bright area corresponds to a magnetization pointing away from the substrate, which corresponds to the area where the magnetization was reversed due to the applied field pulse, and the dark area to the not reversed magnetization. Bright and dark areas are delimited by a single DW localized at places with similar *H*_C_ (Figure 4b). Because in the investigated sample the *H*_C_ gradient exists both in the *x*- and *y*-directions (Figure 1b), the increase of the amplitude of the magnetic field pulses will result in a DW propagation along both directions.

This process is shown in more details in Figure 3 whose panel (a) displays wall propagation in the *x*-direction and panel (b) in the *y*-direction, and panel (c) shows the mean DW position as a function of field pulse amplitude. The *x*-coordinate of area B (indicated in Figure 4) was chosen such that the DW propagation in both *x*- and *y*-directions could be measured within the same field range. This implies that the applied He^+^ ion fluence range is similar for both discussed areas. The interpretation of the magnetization reversal mechanisms deduced from the magnetic structure images of the two areas A (small coercive field gradient = small gradient in average anisotropy) and B (high coercive field gradient = high gradient in average anisotropy) after the application of consecutive external field pulses with increasing magnitude *H*_ext_ will be discussed for graded anisotropy areas A and B separately. For the low anisotropy gradient area A, magnetization reversal starts for small *H*_ext_ in the area with the lowest *H*_C_, where *H*_ext_ ≈ *H*_C_. This area corresponds to the area bombarded by the high ion fluence. There, many small domains nucleate as seen in Figure 3a (uppermost panel). Their density decreases as *H*_C_ increases along the *x*-coordinate of the stripe.

For this low external field, no straight DW is present. In contrast, a gradual transition from one to the other magnetization state by a gradual increase of the density of nucleated domains and their coalescence over an *x*-coordinate range Δ*x* is observed. In the areas of significantly reduced PMA (low *H*_C_, high *F*), domain nucleation is the dominant magnetization reversal mechanism. The formation of a multidomain state is more likely the closer the fluence causes a perpendicular anisotropy decrease close to the magnetization reorientation transition [27]. Domain nucleation is a signature of inherent lateral fluctuations of the effective anisotropy [2,28,29] in a material with small average anisotropy such that the energetic cost of DW formation is small.

In Figure 3a, the lowermost four panels, a gradual formation of a single nearly straight DW with only a few additionally nucleated domains is obvious for magnetic field pulses of higher *H*_ext_, i.e., in areas bombarded with lower fluences and therefore having higher average *H*_C_. In a material with laterally fluctuating but in average higher *K*_eff_, the energetic cost of DW formation (∝*l*√*K*_eff_*A*, *A* is the exchange stiffness) is higher such that the total lengths *l* of the domain walls in the system will be minimized, leading preferentially to a single straight wall. Therefore, the results shown in Figure 3a indicate that suitably high values of *H*_C_ in the whole graded anisotropy material system are a necessary condition for the magnetization reversal by the propagation of a single DW. The distinct difference in magnetization reversal for areas bombarded with high and low fluences (i.e., characterized by weak and strong anisotropy) is probably responsible for the nonlinear dependences presented in Figure 2.

In Figure 3b, the magnetization reversal in an area with high anisotropy gradient is imaged. For low *H*_ext_ (*H*_ext_ < 21.9 kA/m), the magnetization reversal also takes place by small domain nucleation. However, the coalescence of the domains and formation of a single DW is observed at smaller *H*_ext_ as in area A. Therefore, in contrast to the low anisotropy gradient area A, the reversal process occurs virtually in the whole presented *H*_ext_ range by the propagation of a single, nearly straight DW. The realization of the propagation of a single, straight DW in the direction perpendicular to the *H*_C_ gradient is thus easier in systems with larger gradients. In the high gradient material, the influence of intrinsic fluctuations of anisotropy is hardly visible. It should be remarked that the distance travelled by the DW is in the tenths of millimeter range as required for magnetic particle manipulation [30]. The fact that displacements of DW (Δ*x*_DW_, Δ*y*_DW_), in the range of small fluences, are proportional to the amplitude of the magnetic field pulse is a significant advantage of a system with *H*_C_ gradient (see Figure 3c). This circumstance and a significant (about tenfold) difference of the displacement range of the DW in areas with different *H*_C_ gradients (areas A and B) indicate that DW propagation in our system progresses differently than in areas that were not bombarded. The results shown in Figure 3 indicate, in our opinion, that the coercive field gradient limits thermally activated DW propagation, wherein the effect is stronger the higher d*H*_C_/d*x*(*y*). This is of practical importance because it can be used for precise positioning of a DW with pulses of uniform magnetic field. To quantitatively describe DW motion and to specify its regime of propagation in layered systems with an anisotropy gradient, additional measurements are required. However, based on Figure 3c and remembering that pulse duration equals 1 s, we estimated the domain wall velocity *v*_A_ > 0.6 mm/s within area A and *v*_B_ > 0.05 mm/s within area B in the fields 24 < *H*_ext_ < 32 kA/m. Thus, we can assume that DW propagation most probably occur in the ‘depinning’ regime, i.e., the one intermediate between creep and viscous flow regimes [31].

The images of the domain structure shown in Figures 3 and 4 exhibit only two contrasts (gray levels) indicating that the magnetization reversal in both Co layers takes place simultaneously by the propagation of two parallelly moving DWs in the two ferromagnetic layers which has been achieved by the present choice of the Au layer thickness. This effect, in contrast to the independent magnetization reversal of the individual layers observed in [6], is important for ‘magnetic bead transport’ as much higher stray fields over DWs can be fabricated.

## Conclusions

In summary, a dedicated scheme for magnetic multilayer bombardment with light ions was introduced enabling to realize a defined fluence gradient for bombardment of samples along a given coordinate without necessitating a wedged ion stopper layer. This bombardment scheme was applied to Au/Co/Au/Co/Au multilayers, where it allowed to create in both Co layers an approximately constant gradient in perpendicular anisotropy along a preset direction. This scheme allowed a systematic investigation of the conditions to generate in both ferromagnetic layers straight domain walls which can be simultaneously moved and reproducibly positioned by external field pulses of defined amplitude. Quasi-one-dimensional walls can be generated in areas of relatively high *H*_C_ and d*H*_C_/d*x*. For small *H*_C_ and d*H*_C_/d*x*, magnetization reversal is dominated by domain nucleation caused by lateral anisotropy fluctuations in the layer system. If d*H*_C_/d*x* is larger than these inherent lateral *H*_C_ variations, the domain wall becomes straight. These findings are the basis for generating and controllably moving straight domain walls in laterally graded anisotropy layer systems for magnetic bead transport.

## Abbreviations

DW: domain wall; PMA: perpendicular magnetic anisotropy; P-MOKE: polar magneto-optical Kerr effect.

## Competing interests

The authors declare that they have no competing interests.

## Authors’ contributions

FS and BS planned and supervised the study. MM and PK investigated the magnetic characteristics. BS and DL (supervised by AE) designed and performed the ion bombardment experiments. MM and ZK, supervised by AM, collected the images of the domain structure. FS analyzed the data together with MU. FS and AE have been involved in drafting the manuscript and its revision. All authors discussed the results and read and approved the final manuscript.
